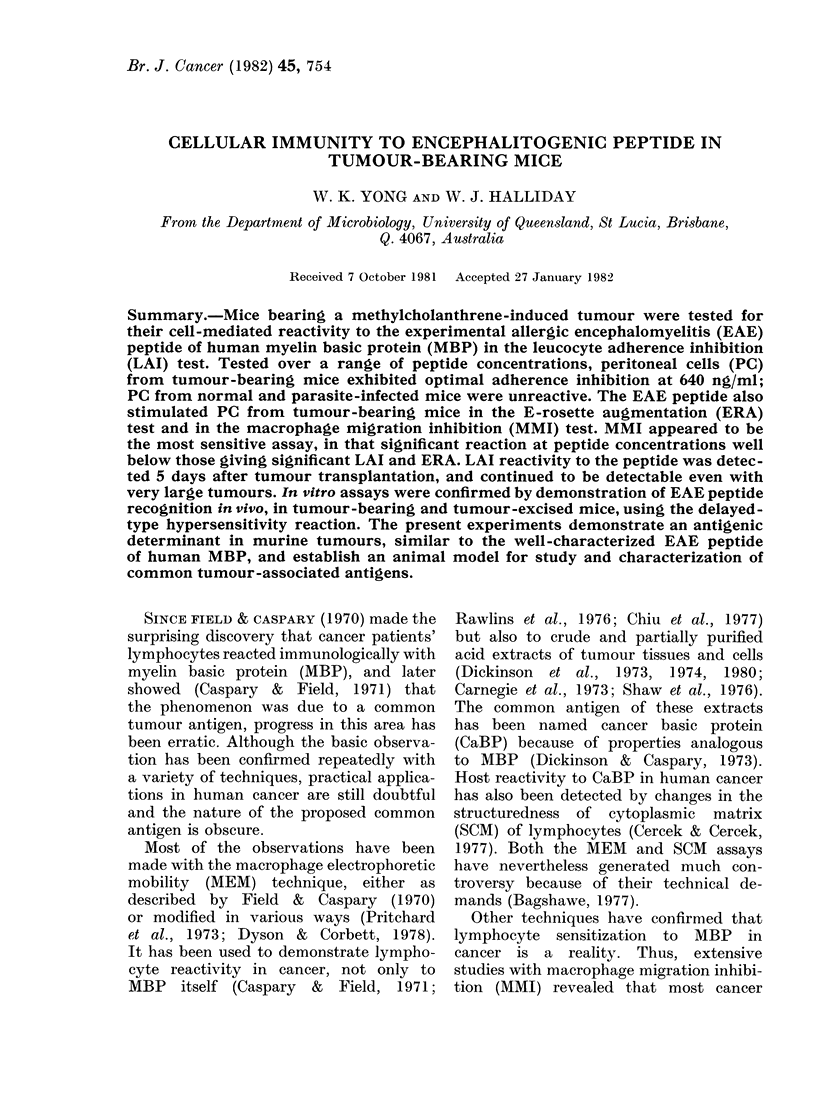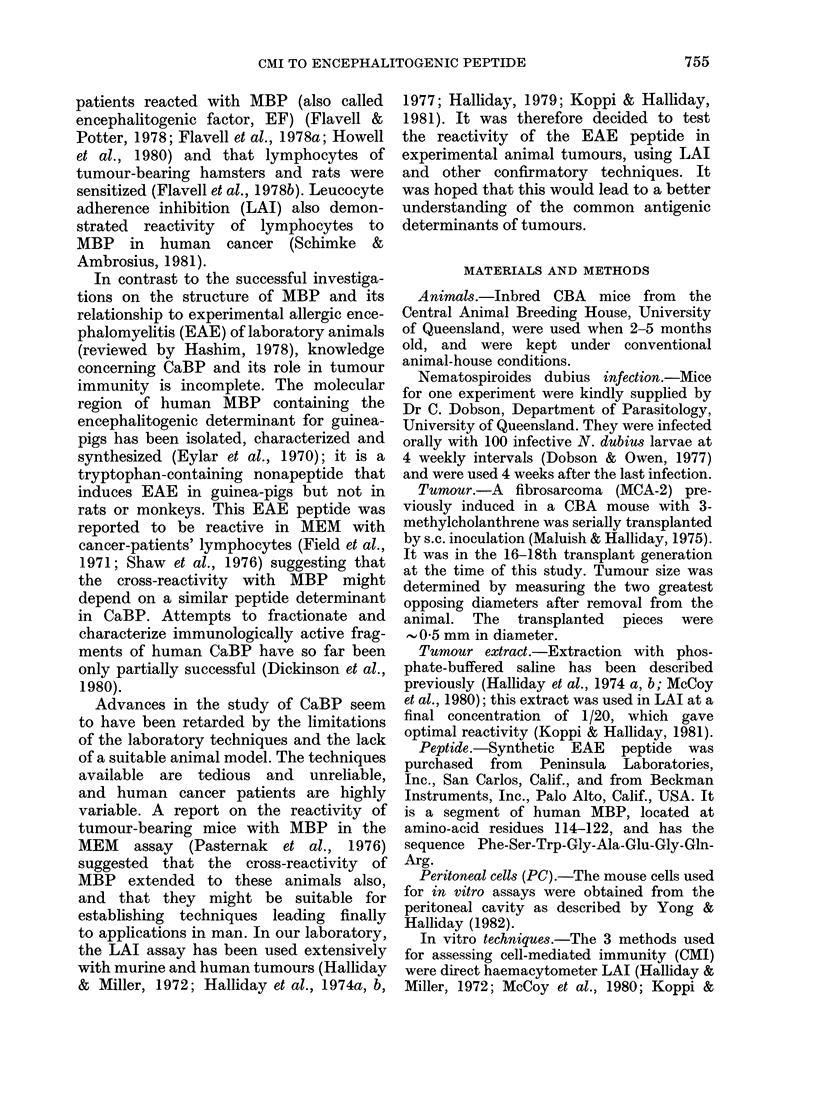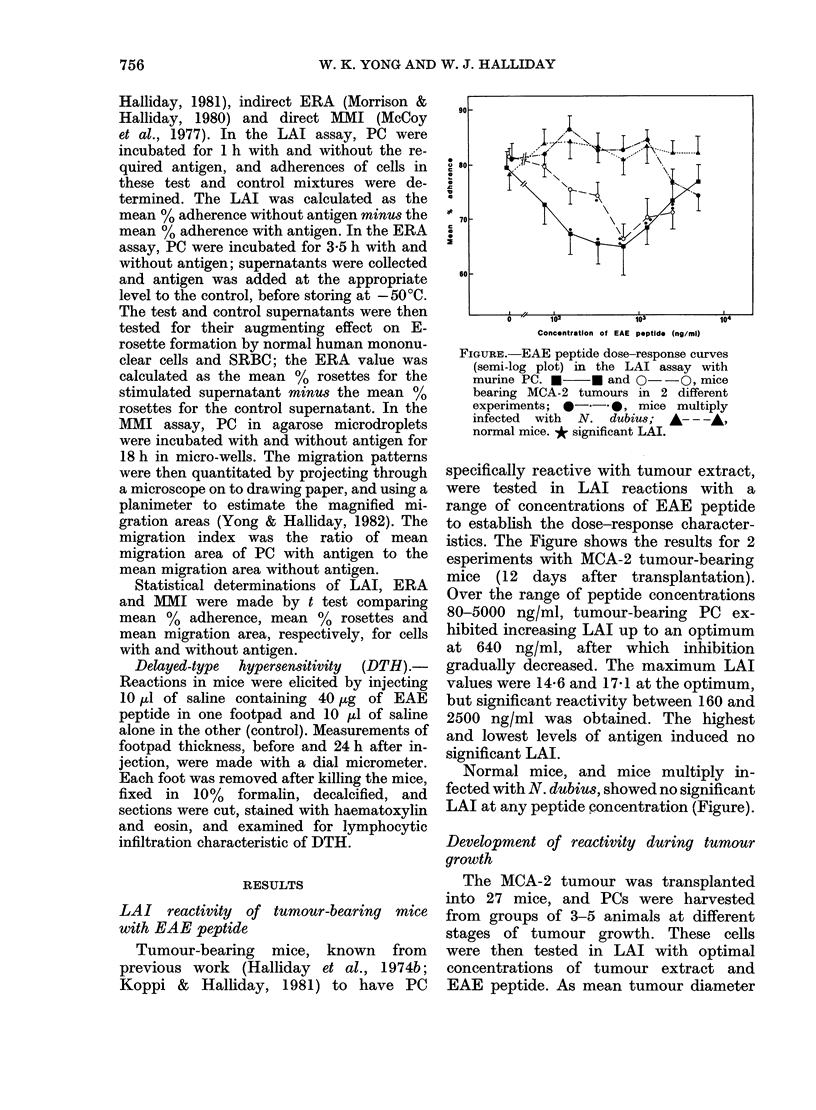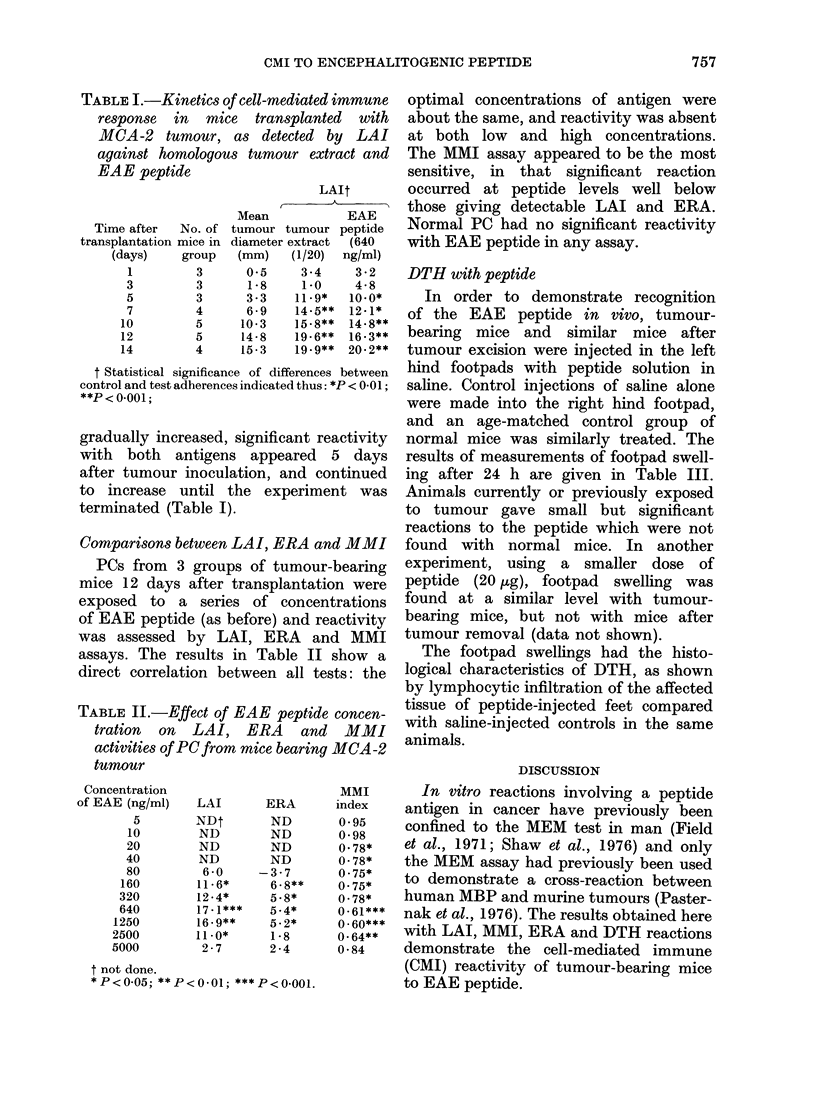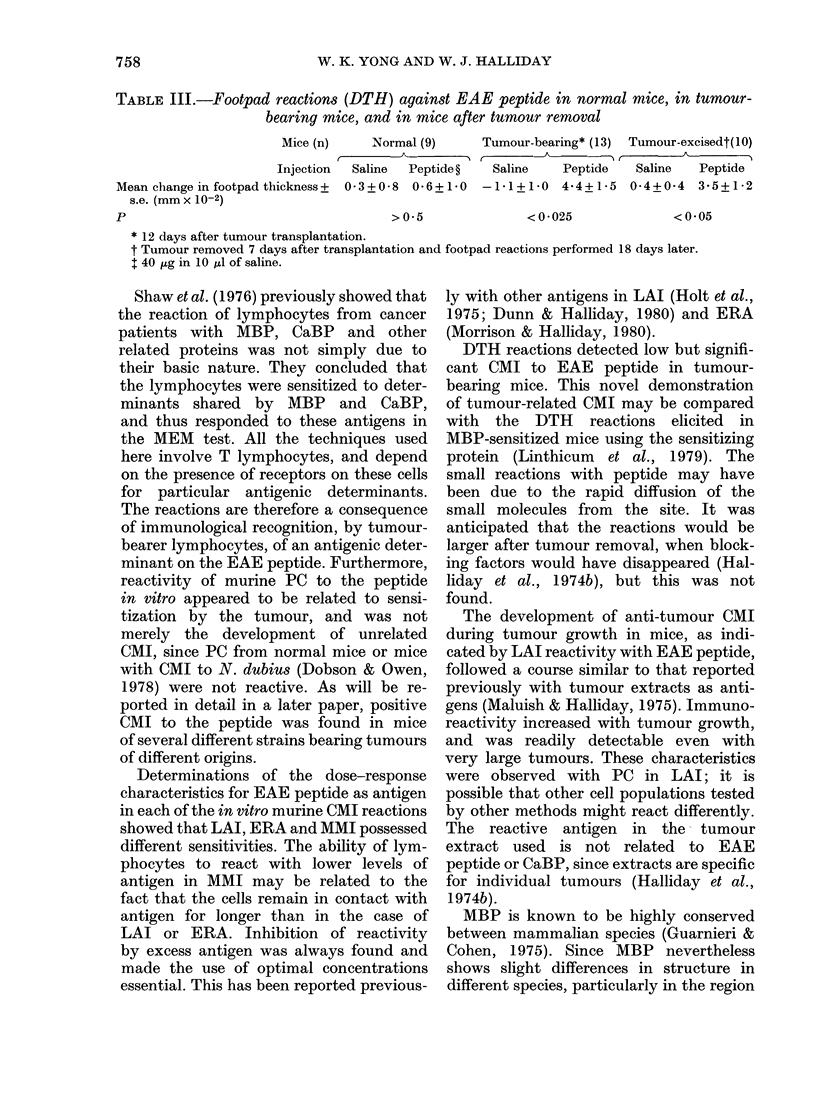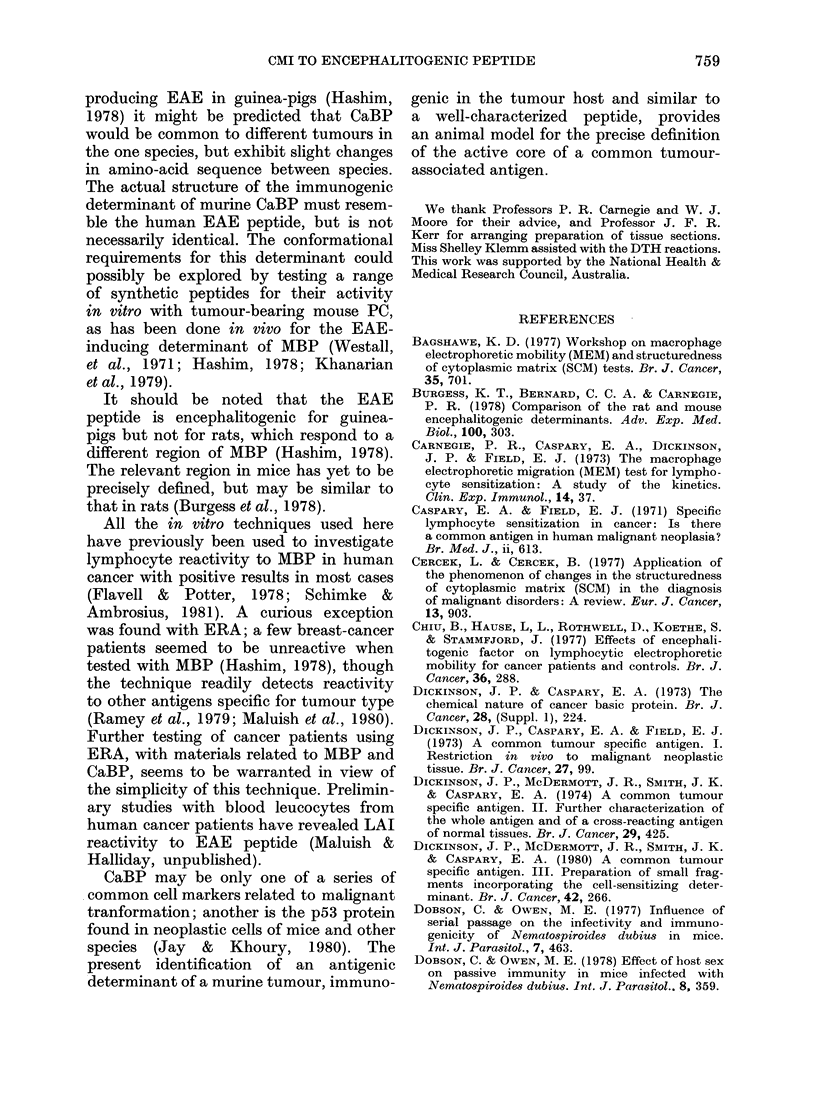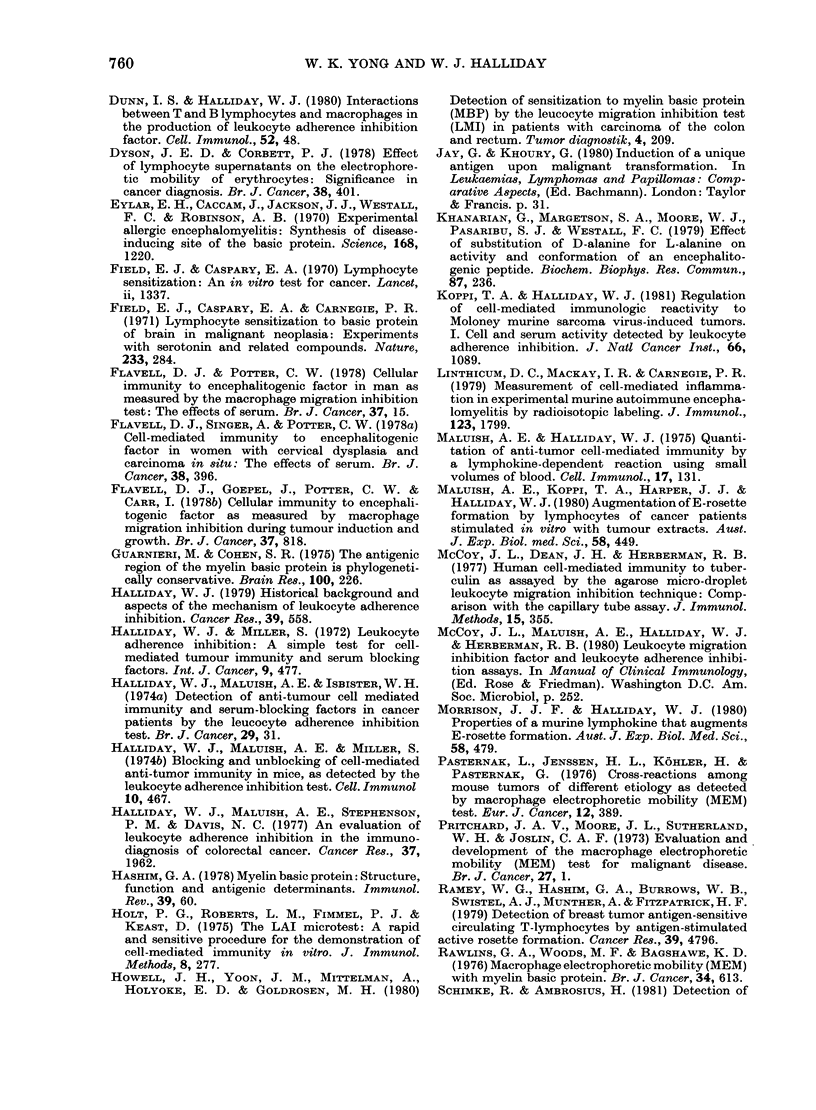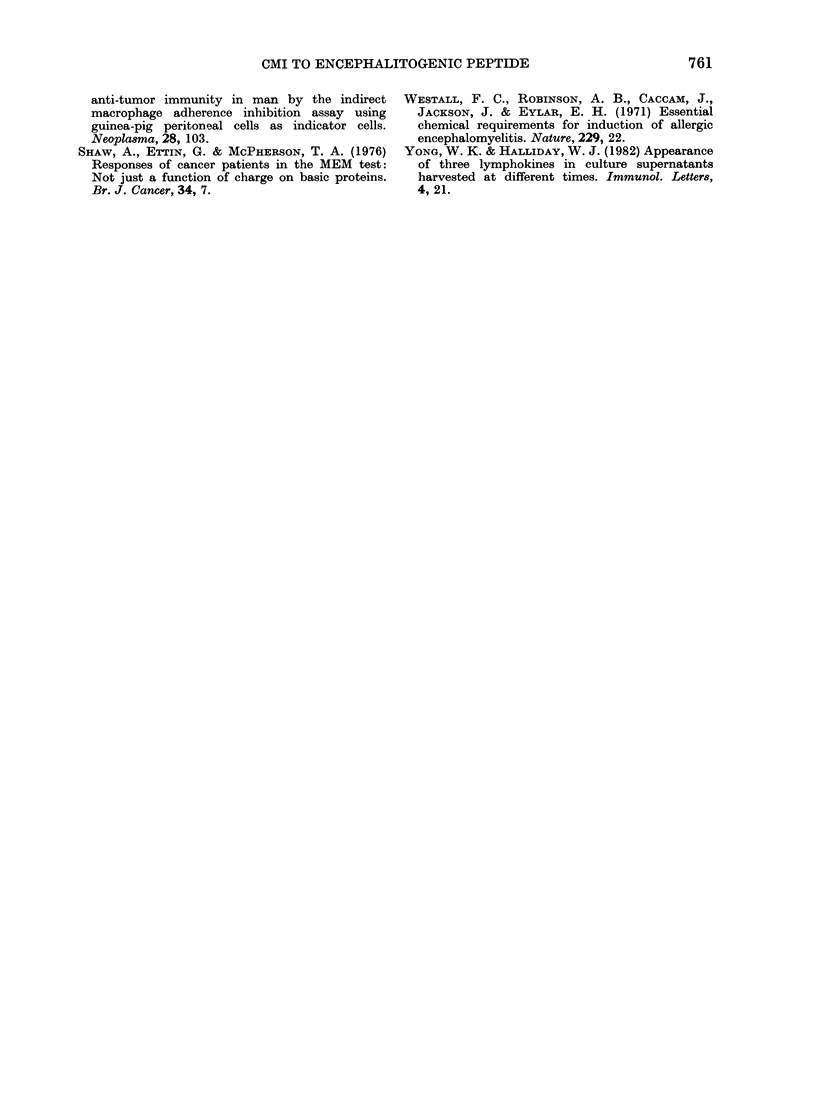# Cellular immunity to encephalitogenic peptide in tumour-bearing mice.

**DOI:** 10.1038/bjc.1982.117

**Published:** 1982-05

**Authors:** W. K. Yong, W. J. Halliday

## Abstract

Mice bearing a methylcholanthrene-induced tumour were tested for their cell mediated reactivity to the experimental allergic encephalomyelitis (EAE) peptide of human myelin basic protein (MBP) in the leucocyte adherence inhibition (LAI) test. Tested over a range of peptide concentrations, peritoneal cells (PC) from tumour-bearing mice exhibited optimal adherence inhibition at 640 ng/ml; PC from normal and parasite-infected mice were unreactive. The EAE peptide also stimulated PC from tumour-bearing mice in the E-rosette augmentation (ERA) test and in the macrophage migration inhibition (MMI) test. MMI appeared to be the most sensitive assay, in that significant reaction at peptide concentrations well below those giving significant LAI and ERA. LAI reactivity to the peptide was detected 5 days after tumour transplantation, and continued to be detectable even with very large tumours. In vitro assays were confirmed by demonstration of EAE peptide recognition in vivo, in tumour-bearing and tumour-excised mice, using the delayed-type hypersensitivity reaction. The present experiments demonstrate an antigenic determinant in murine tumours, similar to the well-characterized EAE peptide of human MBP, and establish an animal model for study and characterization of common tumour-associated antigens.


*Br. J. Cancer* (1982) **45**, 754

### CELLULAR IMMUNITY TO ENCEPHALITOGENIC PEPTIDE IN TUMOUR-BEARING MICE

W. K. YONG AND W. J. HALLIDAY

*From the Department of Microbiology, University of Queensland, St Lucia, Brisbane, Q. 4067, Australia*

Received 7 October 1981 Accepted 27 January 1982

**Summary.—Mice bearing a methylcholanthrene-induced tumour were tested for their cell-mediated reactivity to the experimental allergic encephalomyelitis (EAE) peptide of human myelin basic protein (MBP) in the leucocyte adherence inhibition (LAI) test. Tested over a range of peptide concentrations, peritoneal cells (PC) from tumour-bearing mice exhibited optimal adherence inhibition at 640 ng/ml; PC from normal and parasite-infected mice were unreactive. The EAE peptide also stimulated PC from tumour-bearing mice in the E-rosette augmentation (ERA) test and in the macrophage migration inhibition (MMI) test. MMI appeared to be the most sensitive assay, in that significant reaction at peptide concentrations well below those giving significant LAI and ERA. LAI reactivity to the peptide was detected 5 days after tumour transplantation, and continued to be detectable even with very large tumours. *In vitro* assays were confirmed by demonstration of EAE peptide recognition *in vivo*, in tumour-bearing and tumour-excised mice, using the delayed-type hypersensitivity reaction. The present experiments demonstrate an antigenic determinant in murine tumours, similar to the well-characterized EAE peptide of human MBP, and establish an animal model for study and characterization of common tumour-associated antigens.**

SINCE FIELD & CASPARY (1970) made the surprising discovery that cancer patients' lymphocytes reacted immunologically with myelin basic protein (MBP), and later showed (Caspary & Field, 1971) that the phenomenon was due to a common tumour antigen, progress in this area has been erratic. Although the basic observation has been confirmed repeatedly with a variety of techniques, practical applications in human cancer are still doubtful and the nature of the proposed common antigen is obscure.

Most of the observations have been made with the macrophage electrophoretic mobility (MEM) technique, either as described by Field & Caspary (1970) or modified in various ways (Pritchard *et al.*, 1973; Dyson & Corbett, 1978). It has been used to demonstrate lymphocyte reactivity in cancer, not only to MBP itself (Caspary & Field, 1971; Rawlins *et al.*, 1976; Chiu *et al.*, 1977) but also to crude and partially purified acid extracts of tumour tissues and cells (Dickinson *et al.*, 1973, 1974, 1980; Carnegie *et al.*, 1973; Shaw *et al.*, 1976). The common antigen of these extracts has been named cancer basic protein (CaBP) because of properties analogous to MBP (Dickinson & Caspary, 1973). Host reactivity to CaBP in human cancer has also been detected by changes in the structuredness of cytoplasmic matrix (SCM) of lymphocytes (Cercek & Cercek, 1977). Both the MEM and SCM assays have nevertheless generated much controversy because of their technical demands (Bagshawe, 1977).

Other techniques have confirmed that lymphocyte sensitization to MBP in cancer is a reality. Thus, extensive studies with macrophage migration inhibition (MMI) revealed that most cancer patients reacted with MBP (also called encephalitogenic factor, EF) (Flavell & Potter, 1978; Flavell *et al.*, 1978*a*; Howell *et al.*, 1980) and that lymphocytes of tumour-bearing hamsters and rats were sensitized (Flavell *et al.*, 1978*b*). Leucocyte adherence inhibition (LAI) also demonstrated reactivity of lymphocytes to MBP in human cancer (Schimke & Ambrosius, 1981).

In contrast to the successful investigations on the structure of MBP and its relationship to experimental allergic encephalomyelitis (EAE) of laboratory animals (reviewed by Hashim, 1978), knowledge concerning CaBP and its role in tumour immunity is incomplete. The molecular region of human MBP containing the encephalitogenic determinant for guinea-pigs has been isolated, characterized and synthesized (Eylar *et al.*, 1970); it is a tryptophan-containing nonapeptide that induces EAE in guinea-pigs but not in rats or monkeys. This EAE peptide was reported to be reactive in MEM with cancer-patients' lymphocytes (Field *et al.*, 1971; Shaw *et al.*, 1976) suggesting that the cross-reactivity with MBP might depend on a similar peptide determinant in CaBP. Attempts to fractionate and characterize immunologically active fragments of human CaBP have so far been only partially successful (Dickinson *et al.*, 1980).

Advances in the study of CaBP seem to have been retarded by the limitations of the laboratory techniques and the lack of a suitable animal model. The techniques available are tedious and unreliable, and human cancer patients are highly variable. A report on the reactivity of tumour-bearing mice with MBP in the MEM assay (Pasternak *et al.*, 1976) suggested that the cross-reactivity of MBP extended to these animals also, and that they might be suitable for establishing techniques leading finally to applications in man. In our laboratory, the LAI assay has been used extensively with murine and human tumours (Halliday & Miller, 1972; Halliday *et al.*, 1974*a*, *b*, 1977; Halliday, 1979; Koppi & Halliday, 1981). It was therefore decided to test the reactivity of the EAE peptide in experimental animal tumours, using LAI and other confirmatory techniques. It was hoped that this would lead to a better understanding of the common antigenic determinants of tumours.

### MATERIALS AND METHODS

*Animals.*—Inbred CBA mice from the Central Animal Breeding House, University of Queensland, were used when 2–5 months old, and were kept under conventional animal-house conditions.

Nematospiroides dubius *infection.*—Mice for one experiment were kindly supplied by Dr C. Dobson, Department of Parasitology, University of Queensland. They were infected orally with 100 infective *N. dubius* larvae at 4 weekly intervals (Dobson & Owen, 1977) and were used 4 weeks after the last infection.

*Tumour.*—A fibrosarcoma (MCA-2) previously induced in a CBA mouse with 3-methylcholanthrene was serially transplanted by s.c. inoculation (Maluish & Halliday, 1975). It was in the 16–18th transplant generation at the time of this study. Tumour size was determined by measuring the two greatest opposing diameters after removal from the animal. The transplanted pieces were ~0·5 mm in diameter.

*Tumour extract.*—Extraction with phosphate-buffered saline has been described previously (Halliday *et al.*, 1974 *a*, *b;* McCoy *et al.*, 1980); this extract was used in LAI at a final concentration of 1/20, which gave optimal reactivity (Koppi & Halliday, 1981).

*Peptide.*—Synthetic EAE peptide was purchased from Peninsula Laboratories, Inc., San Carlos, Calif., and from Beckman Instruments, Inc., Palo Alto, Calif., USA. It is a segment of human MBP, located at amino-acid residues 114–122, and has the sequence Phe-Ser-Trp-Gly-Ala-Glu-Gly-Gln-Arg.

*Peritoneal cells (PC).*—The mouse cells used for *in vitro* assays were obtained from the peritoneal cavity as described by Yong & Halliday (1982).

In vitro *techniques.*—The 3 methods used for assessing cell-mediated immunity (CMI) were direct haemacytometer LAI (Halliday & Miller, 1972; McCoy *et al.*, 1980; Koppi &

Halliday, 1981), indirect ERA (Morrison & Halliday, 1980) and direct MMI (McCoy *et al.*, 1977). In the LAI assay, PC were incubated for 1 h with and without the required antigen, and adherences of cells in these test and control mixtures were determined. The LAI was calculated as the mean % adherence without antigen *minus* the mean % adherence with antigen. In the ERA assay, PC were incubated for 3·5 h with and without antigen; supernatants were collected and antigen was added at the appropriate level to the control, before storing at −50°C. The test and control supernatants were then tested for their augmenting effect on E-rosette formation by normal human mononuclear cells and SRBC; the ERA value was calculated as the mean % rosettes for the stimulated supernatant *minus* the mean % rosettes for the control supernatant. In the MMI assay, PC in agarose microdroplets were incubated with and without antigen for 18 h in micro-wells. The migration patterns were then quantitated by projecting through a microscope on to drawing paper, and using a planimeter to estimate the magnified migration areas (Yong & Halliday, 1982). The migration index was the ratio of mean migration area of PC with antigen to the mean migration area without antigen.

Statistical determinations of LAI, ERA and MMI were made by *t* test comparing mean % adherence, mean % rosettes and mean migration area, respectively, for cells with and without antigen.

*Delayed-type hypersensitivity* (*DTH*).—Reactions in mice were elicited by injecting 10 μl of saline containing 40 μg of EAE peptide in one footpad and 10 μl of saline alone in the other (control). Measurements of footpad thickness, before and 24 h after injection, were made with a dial micrometer. Each foot was removed after killing the mice, fixed in 10% formalin, decalcified, and sections were cut, stained with haematoxylin and eosin, and examined for lymphocytic infiltration characteristic of DTH.

FIGURE.—EAE peptide dose–response curves (semi-log plot) in the LAI assay with murine PC. ■——■ and ○— —○, mice bearing MCA-2 tumours in 2 different experiments; ●—·—·●, mice multiply infected with *N. dubius;* ▲- - -▲, normal mice. ★ significant LAI.

### RESULTS

### *LAI reactivity of tumour-bearing mice with EAE peptide*

Tumour-bearing mice, known from previous work (Halliday *et al.*, 1974*b*; Koppi & Halliday, 1981) to have PC specifically reactive with tumour extract, were tested in LAI reactions with a range of concentrations of EAE peptide to establish the dose–response characteristics. The Figure shows the results for 2 esperiments with MCA-2 tumour-bearing mice (12 days after transplantation). Over the range of peptide concentrations 80–5000 ng/ml, tumour-bearing PC exhibited increasing LAI up to an optimum at 640 ng/ml, after which inhibition gradually decreased. The maximum LAI values were 14·6 and 17·1 at the optimum, but significant reactivity between 160 and 2500 ng/ml was obtained. The highest and lowest levels of antigen induced no significant LAI.

Normal mice, and mice multiply infected with *N. dubius*, showed no significant LAI at any peptide concentration (Figure).

### *Development of reactivity during tumour growth*

The MCA-2 tumour was transplanted into 27 mice, and PCs were harvested from groups of 3–5 animals at different stages of tumour growth. These cells were then tested in LAI with optimal concentrations of tumour extract and EAE peptide. As mean tumour diameter

TABLE I.—*Kinetics of cell-mediated immune response in mice transplanted with MCA-2 tumour, as detected by LAI against homologous tumour extract and EAE peptide*

| | | | LAI† | |
|---|---|---|---|---|
| Time after transplantation (days) | No. of mice in group | Mean tumour diameter (mm) | tumour extract (1/20) | EAE peptide (640 ng/ml) |
| 1 | 3 | 0·5 | 3·4 | 3·2 |
| 3 | 3 | 1·8 | 1·0 | 4·8 |
| 5 | 3 | 3·3 | 11·9* | 10·0* |
| 7 | 4 | 6·9 | 14·5** | 12·1* |
| 10 | 5 | 10·3 | 15·8** | 14·8** |
| 12 | 5 | 14·8 | 19·6** | 16·3** |
| 14 | 4 | 15·3 | 19·9** | 20·2** |

† Statistical significance of differences between control and test adherences indicated thus: $*P < 0{\cdot}01$; $**P < 0{\cdot}001$; gradually increased, significant reactivity with both antigens appeared 5 days after tumour inoculation, and continued to increase until the experiment was terminated (Table I).

*Comparisons between LAI, ERA and MMI*

PCs from 3 groups of tumour-bearing mice 12 days after transplantation were exposed to a series of concentrations of EAE peptide (as before) and reactivity was assessed by LAI, ERA and MMI assays. The results in Table II show a direct correlation between all tests: the optimal concentrations of antigen were about the same, and reactivity was absent at both low and high concentrations. The MMI assay appeared to be the most sensitive, in that significant reaction occurred at peptide levels well below those giving detectable LAI and ERA. Normal PC had no significant reactivity with EAE peptide in any assay.

TABLE II.—*Effect of EAE peptide concentration on LAI, ERA and MMI activities of PC from mice bearing MCA-2 tumour*

| Concentration of EAE (ng/ml) | LAI | ERA | MMI index |
|---|---|---|---|
| 5 | ND† | ND | 0·95 |
| 10 | ND | ND | 0·98 |
| 20 | ND | ND | 0·78* |
| 40 | ND | ND | 0·78* |
| 80 | 6·0 | −3·7 | 0·75* |
| 160 | 11·6* | 6·8** | 0·75* |
| 320 | 12·4* | 5·8* | 0·78* |
| 640 | 17·1*** | 5·4* | 0·61*** |
| 1250 | 16·9** | 5·2* | 0·60*** |
| 2500 | 11·0* | 1·8 | 0·64** |
| 5000 | 2·7 | 2·4 | 0·84 |

† not done.
$*P < 0{\cdot}05$; $**P < 0{\cdot}01$; $***P < 0{\cdot}001$.

*DTH with peptide*

In order to demonstrate recognition of the EAE peptide *in vivo*, tumour-bearing mice and similar mice after tumour excision were injected in the left hind footpads with peptide solution in saline. Control injections of saline alone were made into the right hind footpad, and an age-matched control group of normal mice was similarly treated. The results of measurements of footpad swelling after 24 h are given in Table III. Animals currently or previously exposed to tumour gave small but significant reactions to the peptide which were not found with normal mice. In another experiment, using a smaller dose of peptide (20 $\mu$g), footpad swelling was found at a similar level with tumour-bearing mice, but not with mice after tumour removal (data not shown).

The footpad swellings had the histological characteristics of DTH, as shown by lymphocytic infiltration of the affected tissue of peptide-injected feet compared with saline-injected controls in the same animals.

### DISCUSSION

*In vitro* reactions involving a peptide antigen in cancer have previously been confined to the MEM test in man (Field *et al.*, 1971; Shaw *et al.*, 1976) and only the MEM assay had previously been used to demonstrate a cross-reaction between human MBP and murine tumours (Pasternak *et al.*, 1976). The results obtained here with LAI, MMI, ERA and DTH reactions demonstrate the cell-mediated immune (CMI) reactivity of tumour-bearing mice to EAE peptide.

TABLE III.—*Footpad reactions (DTH) against EAE peptide in normal mice, in tumour-bearing mice, and in mice after tumour removal*

| Mice (n) | Normal (9) | | Tumour-bearing* (13) | | Tumour-excised† (10) | |
|---|---|---|---|---|---|---|
| Injection | Saline | Peptide§ | Saline | Peptide | Saline | Peptide |
| Mean change in footpad thickness ± s.e. (mm × $10^{-2}$) | 0·3±0·8 | 0·6±1·0 | −1·1±1·0 | 4·4±1·5 | 0·4±0·4 | 3·5±1·2 |
| *P* | | >0·5 | | <0·025 | | <0·05 |

* 12 days after tumour transplantation.
† Tumour removed 7 days after transplantation and footpad reactions performed 18 days later.
‡ 40 μg in 10 μl of saline.

Shaw *et al.* (1976) previously showed that the reaction of lymphocytes from cancer patients with MBP, CaBP and other related proteins was not simply due to their basic nature. They concluded that the lymphocytes were sensitized to determinants shared by MBP and CaBP, and thus responded to these antigens in the MEM test. All the techniques used here involve T lymphocytes, and depend on the presence of receptors on these cells for particular antigenic determinants. The reactions are therefore a consequence of immunological recognition, by tumour-bearer lymphocytes, of an antigenic determinant on the EAE peptide. Furthermore, reactivity of murine PC to the peptide *in vitro* appeared to be related to sensitization by the tumour, and was not merely the development of unrelated CMI, since PC from normal mice or mice with CMI to *N. dubius* (Dobson & Owen, 1978) were not reactive. As will be reported in detail in a later paper, positive CMI to the peptide was found in mice of several different strains bearing tumours of different origins.

Determinations of the dose–response characteristics for EAE peptide as antigen in each of the *in vitro* murine CMI reactions showed that LAI, ERA and MMI possessed different sensitivities. The ability of lymphocytes to react with lower levels of antigen in MMI may be related to the fact that the cells remain in contact with antigen for longer than in the case of LAI or ERA. Inhibition of reactivity by excess antigen was always found and made the use of optimal concentrations essential. This has been reported previously with other antigens in LAI (Holt *et al.*, 1975; Dunn & Halliday, 1980) and ERA (Morrison & Halliday, 1980).

DTH reactions detected low but significant CMI to EAE peptide in tumour-bearing mice. This novel demonstration of tumour-related CMI may be compared with the DTH reactions elicited in MBP-sensitized mice using the sensitizing protein (Linthicum *et al.*, 1979). The small reactions with peptide may have been due to the rapid diffusion of the small molecules from the site. It was anticipated that the reactions would be larger after tumour removal, when blocking factors would have disappeared (Halliday *et al.*, 1974*b*), but this was not found.

The development of anti-tumour CMI during tumour growth in mice, as indicated by LAI reactivity with EAE peptide, followed a course similar to that reported previously with tumour extracts as antigens (Maluish & Halliday, 1975). Immunoreactivity increased with tumour growth, and was readily detectable even with very large tumours. These characteristics were observed with PC in LAI; it is possible that other cell populations tested by other methods might react differently. The reactive antigen in the tumour extract used is not related to EAE peptide or CaBP, since extracts are specific for individual tumours (Halliday *et al.*, 1974*b*).

MBP is known to be highly conserved between mammalian species (Guarnieri & Cohen, 1975). Since MBP nevertheless shows slight differences in structure in different species, particularly in the region producing EAE in guinea-pigs (Hashim, 1978) it might be predicted that CaBP would be common to different tumours in the one species, but exhibit slight changes in amino-acid sequence between species. The actual structure of the immunogenic determinant of murine CaBP must resemble the human EAE peptide, but is not necessarily identical. The conformational requirements for this determinant could possibly be explored by testing a range of synthetic peptides for their activity *in vitro* with tumour-bearing mouse PC, as has been done *in vivo* for the EAE-inducing determinant of MBP (Westall, *et al.*, 1971; Hashim, 1978; Khanarian *et al.*, 1979).

It should be noted that the EAE peptide is encephalitogenic for guinea-pigs but not for rats, which respond to a different region of MBP (Hashim, 1978). The relevant region in mice has yet to be precisely defined, but may be similar to that in rats (Burgess *et al.*, 1978).

All the *in vitro* techniques used here have previously been used to investigate lymphocyte reactivity to MBP in human cancer with positive results in most cases (Flavell & Potter, 1978; Schimke & Ambrosius, 1981). A curious exception was found with ERA; a few breast-cancer patients seemed to be unreactive when tested with MBP (Hashim, 1978), though the technique readily detects reactivity to other antigens specific for tumour type (Ramey *et al.*, 1979; Maluish *et al.*, 1980). Further testing of cancer patients using ERA, with materials related to MBP and CaBP, seems to be warranted in view of the simplicity of this technique. Preliminary studies with blood leucocytes from human cancer patients have revealed LAI reactivity to EAE peptide (Maluish & Halliday, unpublished).

CaBP may be only one of a series of common cell markers related to malignant tranformation; another is the p53 protein found in neoplastic cells of mice and other species (Jay & Khoury, 1980). The present identification of an antigenic determinant of a murine tumour, immunogenic in the tumour host and similar to a well-characterized peptide, provides an animal model for the precise definition of the active core of a common tumour-associated antigen.

We thank Professors P. R. Carnegie and W. J. Moore for their advice, and Professor J. F. R. Kerr for arranging preparation of tissue sections. Miss Shelley Klemm assisted with the DTH reactions. This work was supported by the National Health & Medical Research Council, Australia.

### REFERENCES

Bagshawe, K. D. (1977) Workshop on macrophage electrophoretic mobility (MEM) and structuredness of cytoplasmic matrix (SCM) tests. *Br. J. Cancer*, **35**, 701.

Burgess, K. T., Bernard, C. C. A. & Carnegie, P. R. (1978) Comparison of the rat and mouse encephalitogenic determinants. *Adv. Exp. Med. Biol.*, **100**, 303.

Carnegie, P. R., Caspary, E. A., Dickinson, J. P. & Field, E. J. (1973) The macrophage electrophoretic migration (MEM) test for lymphocyte sensitization: A study of the kinetics. *Clin. Exp. Immunol.*, **14**, 37.

Caspary, E. A. & Field, E. J. (1971) Specific lymphocyte sensitization in cancer: Is there a common antigen in human malignant neoplasia? *Br. Med. J.*, ii, 613.

Cercek, L. & Cercek, B. (1977) Application of the phenomenon of changes in the structuredness of cytoplasmic matrix (SCM) in the diagnosis of malignant disorders: A review. *Eur. J. Cancer*, **13**, 903.

Chiu, B., Hause, L, L., Rothwell, D., Koethe, S. & Stammfjord, J. (1977) Effects of encephalitogenic factor on lymphocytic electrophoretic mobility for cancer patients and controls. *Br. J. Cancer*, **36**, 288.

Dickinson, J. P. & Caspary, E. A. (1973) The chemical nature of cancer basic protein. *Br. J. Cancer*, **28**, (Suppl. 1), 224.

Dickinson, J. P., Caspary, E. A. & Field, E. J. (1973) A common tumour specific antigen. I. Restriction *in vivo* to malignant neoplastic tissue. *Br. J. Cancer*, **27**, 99.

Dickinson, J. P., McDermott, J. R., Smith, J. K. & Caspary, E. A. (1974) A common tumour specific antigen. II. Further characterization of the whole antigen and of a cross-reacting antigen of normal tissues. *Br. J. Cancer*, **29**, 425.

Dickinson, J. P., McDermott, J. R., Smith, J. K. & Caspary, E. A. (1980) A common tumour specific antigen. III. Preparation of small fragments incorporating the cell-sensitizing determinant. *Br. J. Cancer*, **42**, 266.

Dobson, C. & Owen, M. E. (1977) Influence of serial passage on the infectivity and immunogenicity of *Nematospiroides dubius* in mice. *Int. J. Parasitol.*, **7**, 463.

Dobson, C. & Owen, M. E. (1978) Effect of host sex on passive immunity in mice infected with *Nematospiroides dubius*. *Int. J. Parasitol.*, **8**, 359.

DUNN, I. S. & HALLIDAY, W. J. (1980) Interactions between T and B lymphocytes and macrophages in the production of leukocyte adherence inhibition factor. *Cell. Immunol.*, **52**, 48.

DYSON, J. E. D. & CORBETT, P. J. (1978) Effect of lymphocyte supernatants on the electrophoretic mobility of erythrocytes: Significance in cancer diagnosis. *Br. J. Cancer*, **38**, 401.

EYLAR, E. H., CACCAM, J., JACKSON, J. J., WESTALL, F. C. & ROBINSON, A. B. (1970) Experimental allergic encephalomyelitis: Synthesis of disease-inducing site of the basic protein. *Science*, **168**, 1220.

FIELD, E. J. & CASPARY, E. A. (1970) Lymphocyte sensitization: An *in vitro* test for cancer. *Lancet*, ii, 1337.

FIELD, E. J., CASPARY, E. A. & CARNEGIE, P. R. (1971) Lymphocyte sensitization to basic protein of brain in malignant neoplasia: Experiments with serotonin and related compounds. *Nature*, **233**, 284.

FLAVELL, D. J. & POTTER, C. W. (1978) Cellular immunity to encephalitogenic factor in man as measured by the macrophage migration inhibition test: The effects of serum. *Br. J. Cancer*, **37**, 15.

FLAVELL, D. J., SINGER, A. & POTTER, C. W. (1978*a*) Cell-mediated immunity to encephalitogenic factor in women with cervical dysplasia and carcinoma *in situ:* The effects of serum. *Br. J. Cancer*, **38**, 396.

FLAVELL, D. J., GOEPEL, J., POTTER, C. W. & CARR, I. (1978*b*) Cellular immunity to encephalitogenic factor as measured by macrophage migration inhibition during tumour induction and growth. *Br. J. Cancer*, **37**, 818.

GUARNIERI, M. & COHEN, S. R. (1975) The antigenic region of the myelin basic protein is phylogenetically conservative. *Brain Res.*, **100**, 226.

HALLIDAY, W. J. (1979) Historical background and aspects of the mechanism of leukocyte adherence inhibition. *Cancer Res.*, **39**, 558.

HALLIDAY, W. J. & MILLER, S. (1972) Leukocyte adherence inhibition: A simple test for cell-mediated tumour immunity and serum blocking factors. *Int. J. Cancer*, **9**, 477.

HALLIDAY, W. J., MALUISH, A. E. & ISBISTER, W. H. (1974*a*) Detection of anti-tumour cell mediated immunity and serum-blocking factors in cancer patients by the leucocyte adherence inhibition test. *Br. J. Cancer*, **29**, 31.

HALLIDAY, W. J., MALUISH, A. E. & MILLER, S. (1974*b*) Blocking and unblocking of cell-mediated anti-tumor immunity in mice, as detected by the leukocyte adherence inhibition test. *Cell. Immunol* **10**, 467.

HALLIDAY, W. J., MALUISH, A. E., STEPHENSON, P. M. & DAVIS, N. C. (1977) An evaluation of leukocyte adherence inhibition in the immunodiagnosis of colorectal cancer. *Cancer Res.*, **37**, 1962.

HASHIM, G. A. (1978) Myelin basic protein: Structure, function and antigenic determinants. *Immunol. Rev.*, **39**, 60.

HOLT, P. G., ROBERTS, L. M., FIMMEL, P. J. & KEAST, D. (1975) The LAI microtest: A rapid and sensitive procedure for the demonstration of cell-mediated immunity *in vitro. J. Immunol. Methods*, **8**, 277.

HOWELL, J. H., YOON, J. M., MITTELMAN, A., HOLYOKE, E. D. & GOLDROSEN, M. H. (1980) Detection of sensitization to myelin basic protein (MBP) by the leucocyte migration inhibition test (LMI) in patients with carcinoma of the colon and rectum. *Tumor diagnostik*, **4**, 209.

JAY, G. & KHOURY, G. (1980) Induction of a unique antigen upon malignant transformation. In *Leukaemias, Lymphomas and Papillomas: Comparative Aspects*, (Ed. Bachmann). London: Taylor & Francis. p. 31.

KHANARIAN, G., MARGETSON, S. A., MOORE, W. J., PASARIBU, S. J. & WESTALL, F. C. (1979) Effect of substitution of D-alanine for L-alanine on activity and conformation of an encephalitogenic peptide. *Biochem. Biophys. Res. Commun.*, **87**, 236.

KOPPI, T. A. & HALLIDAY, W. J. (1981) Regulation of cell-mediated immunologic reactivity to Moloney murine sarcoma virus-induced tumors. I. Cell and serum activity detected by leukocyte adherence inhibition. *J. Natl Cancer Inst.*, **66**, 1089.

LINTHICUM, D. C., MACKAY, I. R. & CARNEGIE, P. R. (1979) Measurement of cell-mediated inflammation in experimental murine autoimmune encephalomyelitis by radioisotopic labeling. *J. Immunol.*, **123**, 1799.

MALUISH, A. E. & HALLIDAY, W. J. (1975) Quantitation of anti-tumor cell-mediated immunity by a lymphokine-dependent reaction using small volumes of blood. *Cell. Immunol.*, **17**, 131.

MALUISH, A. E., KOPPI, T. A., HARPER, J. J. & HALLIDAY, W. J. (1980) Augmentation of E-rosette formation by lymphocytes of cancer patients stimulated *in vitro* with tumour extracts. *Aust. J. Exp. Biol. med. Sci.*, **58**, 449.

MCCOY, J. L., DEAN, J. H. & HERBERMAN, R. B. (1977) Human cell-mediated immunity to tuberculin as assayed by the agarose micro-droplet leukocyte migration inhibition technique: Comparison with the capillary tube assay. *J. Immunol. Methods*, **15**, 355.

MCCOY, J. L., MALUISH, A. E., HALLIDAY, W. J. & HERBERMAN, R. B. (1980) Leukocyte migration inhibition factor and leukocyte adherence inhibition assays. In *Manual of Clinical Immunology*, (Ed. Rose & Friedman). Washington D.C. Am. Soc. Microbiol, p. 252.

MORRISON, J. J. F. & HALLIDAY, W. J. (1980) Properties of a murine lymphokine that augments E-rosette formation. *Aust. J. Exp. Biol. Med. Sci.*, **58**, 479.

PASTERNAK, L., JENSSEN, H. L., KÖHLER, H. & PASTERNAK, G. (1976) Cross-reactions among mouse tumors of different etiology as detected by macrophage electrophoretic mobility (MEM) test. *Eur. J. Cancer*, **12**, 389.

PRITCHARD, J. A. V., MOORE, J. L., SUTHERLAND, W. H. & JOSLIN, C. A. F. (1973) Evaluation and development of the macrophage electrophoretic mobility (MEM) test for malignant disease. *Br. J. Cancer*, **27**, 1.

RAMEY, W. G., HASHIM, G. A., BURROWS, W. B., SWISTEL, A. J., MUNTHER, A. & FITZPATRICK, H. F. (1979) Detection of breast tumor antigen-sensitive circulating T-lymphocytes by antigen-stimulated active rosette formation. *Cancer Res.*, **39**, 4796.

RAWLINS, G. A., WOODS, M. F. & BAGSHAWE, K. D. (1976) Macrophage electrophoretic mobility (MEM) with myelin basic protein. *Br. J. Cancer*, **34**, 613.

SCHIMKE, R. & AMBROSIUS, H. (1981) Detection of anti-tumor immunity in man by the indirect macrophage adherence inhibition assay using guinea-pig peritoneal cells as indicator cells. *Neoplasma*, **28**, 103.

Shaw, A., Ettin, G. & McPherson, T. A. (1976) Responses of cancer patients in the MEM test: Not just a function of charge on basic proteins. *Br. J. Cancer*, **34**, 7.

Westall, F. C., Robinson, A. B., Caccam, J., Jackson, J. & Eylar, E. H. (1971) Essential chemical requirements for induction of allergic encephalomyelitis. *Nature*, **229**, 22.

Yong, W. K. & Halliday, W. J. (1982) Appearance of three lymphokines in culture supernatants harvested at different times. *Immunol. Letters*, **4**, 21.